# Oral mucosal findings in ambulatory patients with inflammatory bowel disease

**DOI:** 10.1590/1807-3107bor-2025.vol39.095

**Published:** 2025-09-15

**Authors:** Lilly ESQUIVEL-PEDRAZA, Laura FERNÁNDEZ-CUEVAS, Isianel DELGADO-MARTÍNEZ, Alba CICERO-CASARRUBIAS, María del Pilar MILKE-GARCÍA, Eire Mairan CHANG-BOOL, Linda Margarita BARRAGÁN-HEREDIA, Jenny MALDONADO-MOLINA, Renata Lucrecia RIVERA-FLORES, Jesús Kazuo YAMAMOTO-FURUSHO, Judith DOMÍNGUEZ-CHERIT, Silvia MÉNDEZ-FLORES

**Affiliations:** (a) Instituto Nacional de Ciencias Médicas y Nutrición “Salvador Zubirán”, Department of Dermatology, Mexico City, Mexico.; (b) Centro Dermatológico “Dr. Ladislao de la Pascua”, Mouth Diseases Clinic, Mexico City, Mexico.; (c) Hospital Shriners, Clinical Nutrition, Mexico City, Mexico.; (d) Instituto Nacional de Ciencias Médicas y Nutrición “Salvador Zubirán”, Department of Immunology and Rheumatology, Mexico City, Mexico.; (e) Instituto Nacional de Ciencias Médicas y Nutrición “Salvador Zubirán”, Department of Hematologic Oncology, Mexico City, Mexico.; (f) Instituto Nacional de Ciencias Médicas y Nutrición “Salvador Zubirán”, Division of Nutrition, Mexico City, Mexico.; (g) Inbody Mexico, Clinical Research, Mexico City, Mexico.; (h) Instituto Nacional de Ciencias Médicas y Nutrición “Salvador Zubirán”, Department of Gastroenterology, Mexico City, Mexico.

**Keywords:** Inflammatory Bowel Disease, Crohn Disease, Colitis, Ulcerative, Mouth Mucosa

## Abstract

A cross-sectional study was carried out among patients with ulcerative colitis (UC) and Crohn’s disease (CD) in order to determine the frequency of oral mucosal lesions or conditions (OL), as well as to analyze its relationship with some clinical and laboratory parameters. Epidemiologic, clinical, and laboratory data were considered. Statistics included univariate and multivariate analyses. Ninety patients [46 (51.1%) males] were included [median age: 43 years (range 18–79 years)]. UC was diagnosed in 65 (72.2%) patients; CD in 25 (27.8%) patients; and inactive CD was detected in 78 (86.6%) patients. All patients (100%) had OL; fissured tongue was the most frequent finding [68 (75.6%)]. Furred tongue was more common in UC than in CD patients [45 (69.2%) vs. 11(44.0%); p = 0.03]; lower levels of hemoglobin were more often detected in mucosal pallor [(median (Md) =12.1 vs. 14.4_g/dL)_; p = 0.02] than in other OLs. Higher frequency of melanosis was observed when oral rinses were used [37 (71.2%) vs. 15 (28.8%)]; p = 0.03], compared to those who did not use them. A higher risk of varix [OR: = 37.6 (95%CI: 4.7–298.9), p < 0.001], leukoedema [OR: 5.8 (95%CI: 1.4–24.2); p = 0.004], candidosis [OR: 3.9 (95%CI: 1.4–10.6); p = 0.05], fissured tongue [OR: 3.8 (95%CI: 1.2–11.5); p = 0.01], and all infectious processes analyzed collectively [OR: 3.6 (95%CI: 1.3–9.8); p = 0.03], was found in patients older than 45 years than in younger ones. Also, patients with fissured tongue presented a higher risk of having candidosis than those without this condition [OR: 6.1 (95%CI: 2.1–17.5); p = 0.007]. OLs were highly frequently observed in UC and CD patients. Age (> 45 years), low levels of hemoglobin, use of mouthwashes, among other variables, were predictive factors of OL in these patients; thus, their assessment and detection in inflammatory bowel disease should be emphasized.

## Introduction

Inflammatory bowel disease (IBD) is a group of inflammatory conditions of the colon and small intestine. Ulcerative colitis (UC) and Crohn’s disease (CD) are the main types of IBD. UC is restricted to the colon, but CD can affect the entire gastrointestinal tract, from the mouth to the anus.^1^

The mouth is frequently affected in IBD, with a wide range of mucosal manifestations arising from various pathophysiological mechanisms. Thus, it is important to become acquainted with oral mucosal lesions or conditions (OLs) in IBD because some may exacerbate the underlying condition or interfere with the patient’s quality of life or with treatment.

Different oral manifestations have been described in IBD. Cobblestoning, indurated tag-like lesions, mucogingivitis, lip swelling with vertical fissures, deep linear ulceration, and edema, have been considered by some authors as specific oral lesions in IBD, while recurrent aphthous stomatitis, pyostomatitis vegetans, angular cheilitis, glossitis, periodontitis, perioral dermatitis, recurrent abscesses, deep oral fissuring, submandibular lymphoadenopathy, and salivary duct fistula have been described as non-specific oral lesions in IBD.^2-4^These clinical signs can precede intestinal involvement,^5^ but are, nonetheless, hardly detected by physicians.^2^Moreover, most of the published work is a compilation of the oral lesions that can be observed in IBD, but prevalence has been reported only for the most common lesions.

In Mexico, the impact of IBD on the oral mucosa has been insufficiently studied and existing information remains largely limited. Therefore, the objectives of this study were to find the frequency and characteristics of OLs observed in patients with CD and UC, as well as to assess their association with variables such as sex, age, nutritional status, and laboratory findings, in a reference clinic for IBD patients at a tertiary care center specializing in nutrition, in Mexico City.

## Methodology

A cross-sectional analytical study was performed in consecutive patients diagnosed with UC or CD, who attended the outpatient-IBD clinic of the Department of Gastroenterology, at the Instituto Nacional de Ciencias Médicas y Nutrición “Salvador Zubirán” (INCMNSZ) between 2016 and 2023, in order to determine the frequency of OLs in IBD patients, in addition analyzing their relationship with age, sex, nutritional status (anthropometric measurements and food intake), disease activity, drugs, and laboratory variables such as complete blood count, serum levels of vitamin B_12_, folic acid, glucose, albumin, and C reactive protein.

The inclusion criteria for the study were individuals aged ≥ 18 years with a confirmed diagnosis of IBD (CD or UC) according to international criteria,^6,7^who were willing to sign the informed consent form. Exclusion criteria involved patients who could not undergo a complete oral examination, as well as patients with a diagnosis of indeterminate colitis who had been subjected to proctocolectomy and/or oncologic patients on chemotherapy.

Candidates from the IBD clinic were identified and invited to participate in the study if they met all the inclusion criteria and none of the exclusion criteria. Sampling was systematic, non-probabilistic, and consecutive. Sixty-nine patients who fulfilled the criteria of interest were randomly selected one month prior to the beginning of this study, based on historical records from the IBD consultation [total population (N)]. The sample size was calculated based on the information outlined above, with a 95% confidence interval (Z) and a margin of error (E) of 0.05. A minimum sample size of 59 patients was obtained. The following equation was used, where p was the proportion of patients with the desired characteristics for the study and q was the number of patients without the desired characteristics:^8,9^


$$n=\frac{Z^{2}pqN}{Ne^{2}+Z^{2}Pq}$$

A systematic oral examination was performed by oral pathology specialists (LEP and LFC). Standardized clinical criteria were used for the diagnosis of oral diseases.^10-12^Clinical diagnoses of oral lesions were confirmed by cytology, histopathology, or laboratory, as required. Smears fixed in alcohol and stained with the periodic acid-*Schiff* technique were taken from the dorsum of the tongue of all patients in order to rule out candidal infection. Also, Papanicolaou-stained smears were routinely taken from oral lesions when herpetic infection was suspected. Xerostomia was clinically assessed as described elsewhere;^13,14^when mucosal dryness was detected, with or without cracked and peeling lips,^13^ and confirmed with a wooden tongue depressor that stuck fast to the oral mucosa.^14^ A photographic record was made of all oral findings.

Blood workup at oral examination included leukocytes, platelets, hemoglobin, mean corpuscular hemoglobin concentration (MCHC), mean corpuscular volume (+ 15 days); serum levels of vitamin B_12_, folic acid, glucose, albumin, and C reactive protein (+ 30 days for inactive patients and ± 1 week for active ones).

All drugs (including antibiotics or antifungals) used 30 days prior to oral examination were considered and obtained from the medical charts. Local factors, including dental, orthodontic or prosthetic cutting edges, daily use of dentures or mouthwash, a history of oral mucosal lesions (including a previous diagnosis of herpetic infection, and self-reported OL prior to hospital admission), smoking and/or drinking and related symptoms, were also considered. Oral pain was graded according to a visual analog scale (VAS). Smoking was categorized as occasional (<5 cigarettes/week), mild (> 1 cigarette/day, < 5 cigarettes/day), moderate (5–19 cigarettes/day), strong (≥ 20 cigarettes/day), or former smoker (at least 3 months without smoking). Drinking was considered as positive (daily ingestion or intoxication at least once a week), occasional (ingestion less than once a day), or negative (including former drinkers) based on the previous six months to the study.

The oral hygiene status was also determined according to the simplified oral hygiene index; arbitrarily, an index ≤ 1.0 was considered good oral hygiene and an index > 1.0 was considered poor oral hygiene.

The nutritional status of patients was evaluated by standardized dietitians (IADM and MPMG). The following anthropometric measurements were taken: weight, height, mid-arm circumference, and triceps skinfold as an indirect measurement of fat mass. Derived indices included body mass index (BMI) and arm muscle area, as an indirect measurement of muscle mass status. Food intake was evaluated with a typical diet model (energy in kilocalories and macronutrients in percentage of total energy intake) and iron, folate, and cobalamin intake through specific semiquantitative food frequency questionnaires (values expressed as percentages of the recommended daily allowance for each nutrient).

The IBD activity was evaluated as mild, moderate, or severe according to the criteria described elsewhere.^15^Mild [mild diarrhea (four or less times a day) with no more than small amounts of macroscopic blood in stools, absence of fever and tachycardia, mild anemia, and an erythrocyte sedimentation rate not exceeding 30 mm/h.]; moderate [between mild and severe]; severe [severe diarrhea (six or more times a day) with macroscopic blood in stools, fever (over 37.5C, or 37.8°C at least two days out of four), tachycardia (>90 heartbeats per minute), anemia (hemoglobin 75% or less (considering recent transfusion), and a markedly elevated erythrocyte sedimentation rate (≥ 30 mm/h)].^15^

### Statistical analysis

Medians for dimensional variables and percentages for categorical variables were obtained. All variables were analyzed. In order to facilitate registration of lesions, some variables were grouped, and dichotomous variables were constructed to simplify the statistical analysis. Smoking was dichotomized into smokers (including former smokers) and non-smokers; drinking included patients with a positive or occasional habit vs. negative habit or former drinkers. In addition, patients with oral pain were grouped as asymptomatic (VAS = 0) or symptomatic (VAS ≥ 1). Oral infectious processes included herpetic lesions, candidosis, warts, hairy leukoplakia, and HIV-related Kaposi’s sarcoma. Oral candidosis was analyzed individually by type of presentation, and as a whole group, including erythematous, pseudomembranous, and angular cheilitis associated with candidosis. Oral melanoses were analyzed as a group and individually, comprising racial or physiologic hyperpigmentation, diffuse hyperpigmentation (including smokers’ melanosis), and melanocytic macules. Individuals without any type of OL were considered controls (non-exposed) and those with some type of OL were regarded as cases (exposed). Age was categorized into > 45 years and ≤ 45 years for the sake of statistical analysis, based on the median age of our study population.

In order to determine associations between OL and all the included variables, X^2^ or Fisher’s exact tests were used. Kruskal-Wallis or Mann-Whitney U nonparametric tests were used for the comparison of laboratory and nutritional values in patients with and without OL, and UC or CD, as deemed appropriate. Also, the X^2^ linear trend test was used for the evaluation of nutritional status. Odds ratios were calculated at 95% confidence intervals (CI) and a multivariate analysis was performed, including confounding variables such as age, sex, type of IBD disease, use of drugs, smoking and drinking, prosthetic denture, oral rinses, cutting edges, poor oral hygiene, all oral lesions, oral symptoms, BMI, dietetic indicators, and all laboratory values, as deemed appropriate. Statistical significance was set at a two-tailed p ≤ 0.05.

The study was previously approved by the local Research Ethics Committee (registration number: 931.2013). Each study participant signed an informed consent form.

## Results

All of the selected individuals met all of the inclusion criteria and none of the exclusion criteria. A total of 90 (100%) individuals were included; 46 (51.1%) were male and 44 (48.9%) were female, with a median age of 43 years (range of 18–79). Sixty-five (72.2%) patients were diagnosed with UC and 25 (27.8%) with CD; most of them [78 (86.6%)] were in the inactive phase of the disease. The demographic, anthropometric, clinical, dietary, and laboratory characteristics of the included patients are detailed in Tables 1 and 2. Significant differences between UC and CD patients could be observed, according to age, previous history of oral lesions, use of removable prosthesis, serum leukocyte and hemoglobin levels, and nutrition status, as assessed through triceps skinfold thickness.

**Table 1:** Clinical characteristics of patients with inflammatory bowel disease.

Characteristics	UC (n =65)		CD (n = 25)		Total (n = 90)		p-value*
	n	%	n	%	n	%	
Sex							
Male	33	50.8	13	52.0	46	51.1	NS
Female	32	49.2	12	48.0	44	48.8	NS
AgeMd/years (range)	39.0	18-74	55.0	19-79	43.5	18-79	0.02
Disease activity							
Inactive	55	84.6	23	92.0	78	86.6	NS
Mild	6	9.2	2	8.0	8	8.8	NS
Moderate	4	6.2	0	0.0	4	4.4	NS
Use of drugs							
Pharmacological therapy	64	98.5	23	92.0	87	96.6	NS
NSAIDs	64	93.8	9	39.1	74	76.7	< 0.001
Immunosuppressants	29	45.3	17	73.9	46	51.1	0.03
Vitamin supplement	16	25.0	7	30.4	23	25.6	NS
Hormones and metabolites	12	18.8	5	21.7	17	18.9	NS
Antacid	9	14.1	3	13.0	12	13.3	NS
Biological drug	4	6.3	3	13.0	7	7.8	NS
Other**	14	21.9	10	43.5	24	26.7	NS
Smoking							
Occasional	8	12.3	1	4.2	9	10.0	NS
Mild	3	4.6	0	0.0	3	3.3	NS
Moderate	1	1.5	0	0.0	1	1.1	NS
Former smoker	16	24.6	7	29.2	23	25.5	NS
Drinking							
Occasional	15	23.1	9	36.0	24	26.6	NS
Strong	3	4.6	2	8.0	5	5.5	NS
Oral rinses use							
Positive	40	61.5	15	60.0	65	72.2	NS
Dental prosthesis							
Fixed	12	18.5	3	12.0	15	16.6	NS
Removable	4	62.0	6	24.0	10	11.1	0.02
Both	1	1.5	2	8.0	3	3.3	NS
Cutting edges							
Positive	34	52.3	13	52.0	47	52.2	NS
Oral hygiene							
Good	37	56.9	13	52.0	50	55.5	NS
Poor	28	43.1	12	48.0	40	44.4	NS
Presence of oral symptoms	14	21.5	9	36.0	23	25.5	NS
History of herpetic lesions							
Positive	20	30.8	8	32.0	28	31.1	NS
History of other oral lesions***							
Positive	3	4.6	8	32.0	11	12.2	0.001

UC: ulcerative colitis; CD: Crohn’s disease; Md: median; NSAIDs: Nonsteroidal anti-inflammatory drugs; *Statistical significance (X2 or Mann-Whitney U tests); **Including antihypertensive, anxiolytics/antidepressants, fatty acids and mineral drugs; ***Uncertain diagnosis.

**Table 2:** Anthropometric, dietetic, and biochemical assessment of patients with inflammatory bowel disease.

Characteristics	Ulcerative colitis (n = 65)		Crohn’s disease (n = 25)		Total (n = 90)		p-value*
	Md	Range	Md	Range	Md	Range	
Anthropometric indicators							
Weight (Kg)	67.0	40.0–99.0	61.8	33.0–85.0	65.7	33.0–99.0	NS
Height (m)	1.6	1.3–1.8	1.6	1.3–1.7	1.6	1.3–1.8	NS
Body mass index (Kg/m^2^)	25.2	15.4–37.0	24.6	15.4–35.1	25.1	15.4–37.0	NS
	n	%	n	%	n	%	
Triceps skinfold thickness							
Depletion	10	15.4	9	36.0	19	21.1	0.04
Normal	45	69.2	14	56.0	59	65.6	NS
Obesity	10	15.4	2	8.0	12	13.3	NS
Total	65	100.0	25	100.0	90	100.0	
Arm muscle area							
Depletion	13	20.0	7	28.0	20	22.2	NS
Low	8	12.3	2	8.0	10	11.1	NS
Normal	35	53.8	14	56.0	49	54.4	NS
High	9	13.8	2	8.0	11	12.2	NS
Total	65	100.0	29	100.0	90	99.9	
	Md	Range	Md	Range	Md	Range	
Dietetic indicators							
Energy intake (Kcal)	1683.2	1021.0–3660.0	1727.1	1003.0–3790.0	1695.4	1003.0–3790.0	NS
Carbohydrate**	59.0	27.0–89.0	55.0	39.0–73.0	58.3	30.0–89.0	NS
Protein**	16.0	7.0–28.0	17.0	10.0–26.0	16.1	7.0–28.0	NS
Fat**	25.0	10.0–48.0	28.0	14.0–48.0	25.6	10.0–48.0	NS
Folic acid intake (mcg)	386.1	45.6–2242.7	448.8	14.6–954.8	411.4	14.6–2242.7	NS
Folic acid^***^ (%)	1.2	1.0–2.9	1.5	1.0–3.0	1.2	1.0–3.0	NS
Vitamin B_12_ intake (mcg)	25.8	1.0–300.2	49.9	1.2–378.6	27.9	1.0–378.6	NS
Vitamin B_12_^***^ (%)	10.7	1.0–125.0	17.2	1.0–126.0	11.1	1.0–126.0	NS
Iron intake (mg)	107.6	7.8–349.6	74.5	4.3–325.3	95.3	4.3–349.6	NS
Iron^***^ (%)	55.0	1.0–100.0	50.0	1.0–100.0	54.5	1.0–100.0	NS
Laboratory values							
Leukocytes _(K/µL)_	7.6	4.0–16.4	5.9	2.6–9.3	7.1	2.6–16.4	0.003
Platelets _(K/uL)_	282.0	104.0–941.0	277.0	194.0–697.0	280.0	104.0–941.0	NS
Glucose _(mg/dL)_	87.0	61.0–184.0	89.0	71.0–204.0	88.0	61.0–204.0	NS
Hemoglobin _(g/dL)_	14.4	8.3–18.1	12.7	7.2–17.2	14.2	7.2–18.1	0.025
Mean corpuscular volume _(fL)_	90.2	28.3–104.7	90.6	65.0–102.3	90.2	28.3–104.7	NS
MCHC _(g/dL)_	33.1	24.2–39.2	33.0	29.1–34.1	33.0	24.2–39.2	NS
Albumin _(g/dL)_	4.3	2.3–6.0	4.3	2.9–5.1	4.3	2.3–6.0	NS
Folic acid _(ng/mL)_	22.6	9.8–50.4	24.6	5.0–50.4	22.9	5.0–50.4	NS
Vitamin B_12 (pg/mL)_	368.0	128.0–7500.0	375.0	85.0–15000.0	369.0	85.0–15000.0	NS
C–reactive protein _(mg/L)_	0.4	0.02–3.9	0.6	0.03–5.8	0.5	0.02–5.8	NS

Md: Median; MCHC: Mean corpuscular hemoglobin concentration; Arm muscle area (percentiles): ≤ 5 muscle mass depletion; > 5–≤ 15 low muscle mass; > 15–≤ 85 normal muscle mass; > 85 muscle mass above average; Triceps skinfold thickness (percentiles): <10, fat mass depletion; 10 – 90, normal adiposity; > 90, excess fat mass; *X^2^ linear trend test or Mann–Whitney U tests statistical significance. NS: not significant; **% total energy intake; ***Recommended daily allowance.

**Figure 1:**
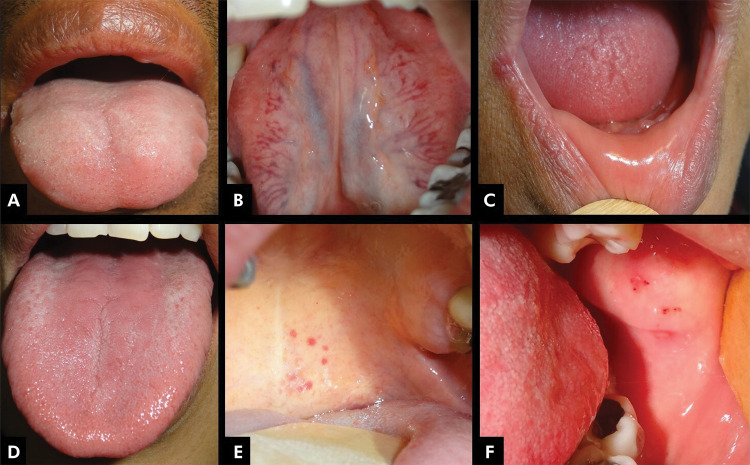
Oral conditions in ulcerative colitis. A: Crenated pale tongue and Fordyce’s spots in vermilion border; B: Ventral tongue varyx; C: Angular cheilitis & xerostomia; D: Typical erythemathous patches on the tongue, due to candidosis; E: Soft palate telangiectasia; F: Traumatic hemorrhagic erosions.

**Figure 2:**
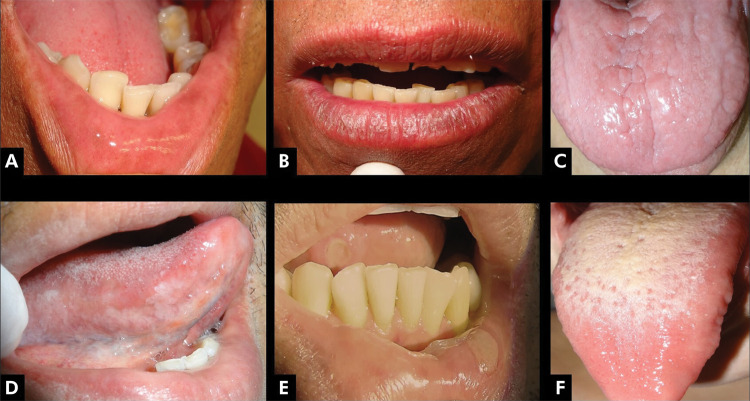
Oral manifestations in Crohn’ disease. A: Melanotic macules in lower lip mucosa; B: Hyperpigmented exfoliative cheilitis; C: Atrophic glossitis, note paleness and subtle erythematous candidosis with fissures, in the middle of the tongue; D: HIV-negative hairy leukoplakia; E: Nutritional deficiency-related aphthous type ulcers; F: Coating tongue.

All patients presented some type of OL (Figures 1 and 2, Table 3). The most frequently observed oral lesions were fissured tongue [68 (75.6%)] and erythematous candidosis [62 (68.9%)]. A higher frequency of candidosis was observed among those patients with fissured tongue in comparison with those without the condition [54 (79.4%) vs. 9 (40.0%); p = 0.001].

**Table 3:** Oral mucosal lesions in patients with inflammatory bowel disease.

Oral lesion^(Affected site)^	Ulcerative colitis (n = 65)		Crohn’s disease (n = 25)		Total (n = 90)		p-value^*^
	n	%	n	%	n	%	
Fissured tongue	48	73.8	20	80.0	68	75.6	NS
Infectious process	44	67.7	20	80.0	64	71.1	NS
*Candidosis*	43	66.2	20	80.0	63	70.0	NS
*Erythematous* ^(^ ^1^ ^,^ ^2^ ^,^ ^5^ ^,^ ^6^ ^,^ ^8^ ^)^	42	64.6	20	80.0	62	68.9	NS
*Pseudomembranous* ^(^ ^1^ ^,^ ^2^ ^,^ ^4^ ^,^ ^6^ ^,^ ^10^ ^)^	5	7.7	1	4.0	6	6.7	NS
*Hairy leukoplakia* ^(^ ^7^ ^)^	1	1.5	1	4.0	2	2.2	NS
*Herpetic lesions* ^(^ ^1^ ^,^ ^10^ ^)^	1	1.5	1	4.0	2	2.2	NS
HPV-related lesion** ^(^ ^6^ ^)^	1	1.5	1	4.0	2	2.2	NS
*HIV-related Kaposi’s sarcoma* ^(^ ^10^ ^)^	0	0.0	1	4.0	1	1.1	NS
Coated tongue	45	69.2	11	44.0	56	62.2	0.03
Melanosis	39	60.0	13	52.0	52	57.8	NS
*Racial hypermelanosis* ^(^ ^1^ ^,^ ^2^ ^,^ ^5^ ^,^ ^6^ ^,^ ^9^ ^,^ ^10^ ^)^	28	43.1	8	32.0	36	40.0	NS
*Melanotic macule* ^(^ ^1^ ^,^ ^2^ ^,^ ^10^ ^)^	14	21.5	3	12.0	17	18.9	NS
*Diffuse hypermelanosis* ^(^ ^1^ ^,^ ^2^ ^,^ ^4^ ^,^ ^6^ ^,^ ^8^ ^,^ ^10^ ^)^	6	9.2	4	16.0	10	11.1	NS
Exfoliative cheilitis	28	43.1	16	64.0	44	48.9	NS
Trauma-related lesions	27	41.5	6	24.0	33	36.7	NS
*Erosion* ^(^ ^1^ ^-^ ^4^ ^,^ ^7^ ^,^ ^9^ ^,^ ^10^ ^)^	18	27.7	5	20.0	23	25.6	NS
*Frictional keratosis* ^(^ ^1^ ^,^ ^2^ ^,^ ^7^ ^)^	5	7.7	0	0.0	5	5.6	NS
*Traumatic ulcer* ^(^ ^1^ ^,^ ^2^ ^,^ ^9^ ^)^	2	3.1	1	4.0	3	3.3	NS
*Morsicatio mucosae oris* ^(^ ^1^ ^,^ ^2^ ^)^	2	3.1	0	0.0	2	2.2	NS
Xerostomia	26	40.0	12	48.0	38	42.0	NS
Atrophic glossitis	22	33.8	14	56.0	36	40.0	NS
Vascular lesions	16	24.6	10	40.0	27	30.0	NS
*Varix* ^(^ ^1^ ^,^ ^2^ ^,^ ^4^ ^,^ ^6^ ^)^	10	15.4	9	36.0	19	21.1	0.04
*Telangiectasia* ^(^ ^1^ ^)^	5	7.7	1	4.0	6	6.7	NS
*Hemangioma* ^(^ ^1^ ^)^	1	1.5	0	0.0	1	1.1	NS
*Venous lake* ^(^ ^1^ ^)^	0	0.0	1	4.0	1	1.1	NS
Crenated tongue	15	23.1	3	12.0	18	20.0	NS
Fordyce spots^(^ ^1^ ^,^ ^2^ ^,^ ^10^ ^)^	13	20.0	3	12.0	16	17.8	NS
Leukoedema^(^ ^1^ ^,^ ^2^ ^,^ ^7^ ^)^	13	20.0	3	12.0	16	17.8	NS
Pallor	8	12.3	5	20.0	13	14.4	NS
Angular cheilitis	9	13.8	4	16.0	13	14.4	NS
Scar^(^ ^1^ ^,^ ^2^ ^,^ ^5^ ^,^ ^6^ ^,^ ^8^ ^,^ ^10^ ^)^	7	10.8	3	12.0	10	11.1	NS
Hemorrhagic lesion ^(^ ^1^ ^,^ ^5^ ^-^ ^7^ ^,^ ^9^ ^)^	6	9.2	1	4.0	7	7.8	NS
White occlusal line (*linea alba*)	6	9.2	1	4.0	7	7.8	NS
Fibrous hyperplasia^(^ ^1^ ^,^ ^2^ ^,^ ^6^ ^,^ ^7^ ^,)^	3	4.6	3	12.0	6	6.7	NS
Benign migratory glossitis	5	7.7	0	0.0	5	5.6	NS
Hairy tongue	4	6.2	1	4.0	5	5.6	NS
Toothpaste-related desquamation^(^ ^1^ ^,^ ^5^ ^)^	3	4.6	2	8.0	5	5.6	NS
Recurrent aphthous ulcers^***(1,2,4,6,7)^	3	4.6	2	8.0	5	5.6	NS
Mucocele^(^ ^1^ ^)^	0	0.0	2	8.0	2	2.2	NS
Other^****^	9	13.8	2	8.0	11	12.2	NS

Statistical significance. X2 or Fisher’s exact tests.**Squamous cell papilloma and Heck’s disease. ***Minor type (three cases), major (one case), and unspecified (one case). **** Erythematous macule(1) (four cases), fissure of the palate, vitiligo spot,(1,10) nevus,(10) lingual papillitis, and amalgam tattoo,(9) in ulcerative colitis. Nutritional deficiency-related ulcers and erythema multiforme in Crohn’s disease. Site: 1=lip, 2=buccal, 3=floor of the mouth, 4=ventral tongue, 5=soft palate, 6=dorsal tongue, 7=lateral border of the tongue, 8=hard palate, 9=gingiva, & 10=vermilion border.

A higher frequency of varyx was observed in CD patients (Table 3) and in those aged over 45 years [18 (94.7%) vs. 1 (5.3%)] in comparison with those aged less than 45 years (p = 0.001); however, in the multivariate analysis (Table 4), only age was significantly related (p < 0.001) after adjusting for confounding variables (type of IBD disease, presence of smoking and drinking habits, cutting edges, oral rinses, candidosis, iron deficiency, use of NSAIDs, or poor oral hygiene).

**Table 4:** Risk factors associated with oral lesions in inflammatory bowel disease.

Risk factor/oral lesion	OR/RR	95% CI	p-value*
Age (> 45 years)			
Varix	37.6	4.7–298.9	< 0.001
Leukoedema	5.8	1.4–24.2	0.004
Candidosis	3.9	1.4–10.6	0.05
Fissured tongue	3.8	1.2–11.5	0.01
All infectious processes^**^	3.6	1.3–9.8	0.03
Sex (Male)			
Fordyce	6.4	1.5–26.5	< 0.001
Leukoedema	3.9	1.2–13.0	0.008
Smoking			
Pseudomembranous candidosis	8.4	1.0–75.1	0.04
Ulcerative colitis			
Coated tongue	1.6	1.0–2.5	0.03
Use of mouthwashes			
Melanosis	1.6	1.0–2.4	0.005
Presence of other oral conditions			
Fissured tongue			
Hairy tongue	12.3	1.4–104.8	0.02
All infectious processes	6.7	2.3–19.2	0.002
Candidosis	6.1	2.1–17.5	0.007
Hairy tongue			
All infectious processes	10.0	1.1–84.0	0.05

* Multinominal logistic regression analysis; **Herpetic lesions, candidosis, warts, hairy leukoplakia, and Kaposi’s sarcoma.

Higher levels of oral melanoses (p = 0.03) were observed in individuals using oral antiseptic mouthwashes [37 (71.2%) vs. 15 (28.8%)] in comparison to those who did not use them; maintaining its significance (p = 0.005) after controlling for confounding factors (age, gender, type of IBD disease, presence of smoking and drinking habits, poor oral hygiene, prosthetic denture, candidosis, ulcers, symptom profile, iron and vitamin B_12_ deficiency, leukopenia, use of NSAIDs, immunosuppressants, vitamin supplements, antacids, low BMI, and dietetic indicators.

Table 4 shows the most relevant associations between the different risk factors analyzed and the detected OL, as well as the associations between them; their statistical significance remained unchanged for all of them, even after adjusting for diverse confounding factors.

The most frequent associations of OLs with the main serological analysis are detailed in Table 5. The analysis of the different serological and nutritional investigations into oral lesions showed that patients with mucosal pallor presented lower levels of hemoglobin (Md = 12.1 vs 14.4 g/dL) than their counterparts (p = 0.02); also, pallor was more frequent in women [11 (84.6%) vs. 2 (15.4%)] and they were at a higher risk [OR: 7.3 (95%CI: 1.5–35.3)] for developing it than men (p = 0.0057).

**Table 5:** Analysis of the main serological findings in IBD patients with the most common oral lesions.

Oral manifestation	Hemoglobin _(g/dL)_		VCM* _(fL)_		Albumin (g/dL)		Folic acid _(ng/mL)_		Vitamin B_12 (pg/mL)_		IBD**
	Md	range	Md	range	Md	range	Md	range	Md	range	
Fissured tongue	14.4	7.2–18.1	90	28–104	4.3	2.3–6.0	22.9	5.1–50.0	328	85–15,000	A
Control	13.0	9.6–17.0	91	78–102	4.2	2.9–5.2	23.3	9.2–50.4	436	174–1,385	
Candidosis	14.3	7.2–18.1	90	28–104	4.3	2.3–5.2	22.6	5.0–50.4	370	85–15,000	B
Control	14.2	9.6–17.2	89	29–104	4.5	2.7–6.0	23.3	9.2–50.4	349	128–6,805	
Coated tongue	14.3	7.2–17.3	90	28–104	4.3	2.3–5.1	24.2	5.3–50.4	370	115–15,000	A
Control	14.0	7.4–18.1	90	32–104	4.4	3.0–6.0	22.6	5.0–50.4	360	85–15,000	
Melanosis	14.2	8.3–18.1	91	29–104	4.4	2.3–5.1	22.4	9.2–50.4	376	150–15,000	C
Control	14.4	7.2–17.7	88	28–104	4.3	2.9–6.0	24.5	5.0–50.4	337	85–1,236	
Exfoliative cheilitis	13.9	7.2–17.3	87	32–99	4.3	2.7–5.2	24.5	5.3–50.4	301	85–15,000	C
Control	14.3	7.4–18.1	91	28–104	4.3	2.3–6.0	22.2	5.0–50.4	384	115–15,000	
Traumatic lesions	13.7	12.5–14.6	92	86–93	4.1	3.5–4.4	22.3	19.4–22.6	236	156–265	B
Control	14.3	7.2–18.1	90	28–104	4.3	2.3–6.0	23.7	5.0–50.4	370	85–15,000	
Xerostomia	14.2	7.2–17.7	90	29–104	4.4	3.0–5.2	23.5	5.0–50.4	386	85–6805	A
Control	14.3	8.3–18.1	90	28–104	4.3	2.3–6.0	22.5	9.2–50.4	341	115–15,000	
Atrophic glossitis	13.6	7.2–16.9	88	32–100	4.2	2.3–4.9	21.9	5.0–50.4	341	85–15,000	A
Control	14.7	9.6–18.1	91	28–104	4.4	3.0–6.0	24.8	9.8–47.3	374	115–15,000	
Varix	14.2	10.8–16.2	92	82–102	4.3	3.0–5.1	25.3	15.2–50.4	368	115–15,000	C
Control	14.3	7.2–18.1	89	28–104	4.3	2.3–6.0	22.4	5.0–50.4	370	85–15,000	
Telangiectasia	14.5	12.3–16.2	96	79–102	4.2	3.6–5.0	25.0	22.3–50.4	442	128–15,000	C
Control	14.2	7.2–18.1	90	28–104	4.3	2.3–6.0	22.6	5.0–50.4	369	85–15,000	
Crenated tongue	14.6	9.6–18.1	93	69–104	4.3	3.6–6.0	22.8	12.4–47.3	413	85–15,000	C
Control	14.2	7.2–17.7	89	28–104	4.3	2.3–5.2	23.1	5.0–50.4	341	128–15,000	
Fordyce spots	14.5	10.1–18.1	82	28–104	4.4	2.7–6.0	21.8	9.2–39.8	384	115–3,476	C
Control	14.0	7.2–17.2	90	30–104	4.3	2.3–5.2	23.8	5.0–50.4	360	85–15,000	
Leukoedema	15.0	11.4–18.1	92	78–104	4.5	3.5–4.9	30.6	9.2–47.3	454	128–1,236	C
Control	14.0	7.2–17.7	90	28–104	4.3	2.3–6.0	22.5	5.0–50.4	350	85–15,000	
Pallor	12.1	7.2–16.9	86	65–102	4.2	2.3–5.0	17.7	5.0–37.5	349	85–1,075	C
Control	14.4	9.6–18.1	90	28–104	4.3	2.7–6.0	23.9	9.2–50.4	370	115–15,000	
Angular cheilitis	13.9	7.4–17.0	87	65–95	4.4	3.0–5.2	22.6	5.0–39.8	436	219–3,476	A
Control	14.3	7.2–18.1	91	28–104	4.3	2.3–6.0	23.5	5.3–50.4	334	85–15,000	
Scar	15.4	12.6–16.8	94	30–97	4.4	3.6–5.1	19.5	10.7–45.2	268	143–517	C
Control	14.0	7.2–18.1	89	28–104	4.3	2.3–6.0	23.8	5.0–50.4	371	85–15,000	
Hemorrhagic lesion	14.0	13.7–16.9	93	79–96	4.3	4.1–4.7	25.3	21.3–33.6	280	128–1,075	B
Control	14.3	7.2–18.1	90	28–104	4.4	2.3–6.0	22.6	5.0–50.4	370	85–15,000	
Occlusal linea alba	13.7	9.6–16.6	89	69–97	4.2	3.9–4.9	22.6	13.9–45.2	439	85–517	C
Control	14.3	7.2–18.1	90	28–104	4.3	2.3–6.0	23.3	5.0–50.4	368	115–15,000	
Fibrous hyperplasia	15.1	13.7–17.0	89	80–93	4.6	4.3–5.2	27.6	17.3–35.1	269	152–6,805	B
Control	14.1	7.2–18.1	90	28–104	4.3	2.3–6.0	22.9	5.0–50.4	370	85–15,000	
Geographic tongue	13.4	12.6–16.3	89	76–100	4.0	3.9–4.6	19.8	9.8–35.1	527	237–7,500	B
Control	14.3	7.2–18.1	90	28–104	4.4	2.3–6.0	23.7	5.0–50.4	368	85–15,000	
Hairy tongue	13.6	11.2–16.6	87	81–94	4.7	2.9–4.7	35.6	15.3–47.3	349	180–1,041	B
Control	14.3	7.2–18.1	90	28–104	4.3	2.3–6.0	22.6	5.0–50.4	370	85–15,000	
Desquamation***	11.4	7.2–16.6	69	29–92	4.2	4.0–4.7	25.2	5.3–47.3	274	85–398	C
Control	14.3	7.4–18.1	90	28–104	4.3	2.3–6.0	22.6	5.0–50.4	370	115–15,000	
Recurrent aphthae	13.9	9.6–17.2	87	81–93	4.1	3.6–4.8	22.3	11.6–23.7	352	174–563	A
Control	14.3	7.2–18.1	90	28–104	4.4	2.3–6.0	23.9	5.0–50.4	370	85–15,000	

*Mean corpuscular volume; *IBD–relationship: A) Unspecific oral lesion (OL) in IBD; B) Other OL described in IBD; C) Not mentioned in IBD; ***Toothpaste–related desquamation.

Patients with angular cheilitis had lower levels of mean corpuscular volume (Md = 87.2 vs. 91.3_fL_; p = 0.03) than individuals without the condition. Those with infectious processes had lower albumin concentrations (Md = 4.3 vs. 4.5_g/dL_; p = 0.03) than their counterparts. A lower energy intake (kcal) was observed in patients with atrophic glossitis (Md = 1473 vs. 1688; p = 0.02) and in those with varyx (Md = 1510 vs, 1660; p =0.04), in comparison with those without these conditions; on the contrary, higher energy intake was observed in patients with exfoliative cheilitis (Md = 1681 vs. 1522; p = 0.02) in contrast to those without this lesion. Nevertheless, in the multivariate analysis, only pallor remained associated with low concentrations of hemoglobin (p = 0.04) and gender (p = 0.03) after adjusting for confounding variables (smoking, type of IBD disease, age, use of antacids, and leukopenia). No other relevant associations were observed in this study.

## Discussion

The study shows the frequency of OLs in IBD at a tertiary care center from Mexico City. This is the first study of its kind in Mexico and the obtained data support the development of preemptive measures and management strategies at our center, which may, in turn, serve as a reference for similar efforts across the country.

The highly anticipated differences between CD and UC in terms of age^16^ and the use of certain drugs^17^ were confirmed in our findings. Even though UC and CD are both associated with weight loss and malnutrition,^6^our study highlights that CD patients presented significantly more cases with a history of oral lesions than those observed among UC patients, a finding that has been scarcely described in the literature. Likewise, the nutritional status of this group was poorer (consistent with lower hemoglobin concentrations and leukocyte counts) than that of UC patients (Table 2). Our findings concur with those of other authors who have noted a greater decline in health among CD patients than among UC patients, primarily due to the disease itself.^18^ This fact could therefore partially explain the greater number of CD patients with previous oral lesions. These findings underscore the need to closely monitor the nutritional status of these patients providing supplementation in case of deficiency.

Most of our patients were in the inactive phase of IBD at the time of assessment; despite this, the frequency of OL was high. The frequency of oral lesions in IBD varies widely in the literature: from 0.5% to 50%.^2^ Most studies have focused on the status of periodontal tissues rather than on mucosal involvement.^2^In our series, the fact that all patients presented some type of condition in the oral mucosa is noteworthy. Fissured tongue, as seen in Table 3, was the most prevalent condition (75.6%), followed by candidosis (70%) and furred tongue (62.2%), contrasting with most reports that describe different types of ulcers as the most frequent finding.^2, 3^Despite the high frequency of these oral conditions, they usually go unnoticed during the oral examination and their presence or prevalence are rarely mentioned in the IBD published data, except for recurrent aphthous ulcers.^19^

In previous reports, methodological aspects such as the involved specialists, study population, IBD type, research objectives, and operational definitions have resulted in an inconsistent frequency of OL and in different descriptions of oral manifestations.^2,3^ In the analysis of 12 studies specifically addressing oral mucosal manifestations in IBD, we found that most oral evaluations were conducted by physicians and dentists, general practitioners, or practitioners from specialties not related to oral pathology (90.7%), with only 9.3% including the participation of oral pathologists.^2^ In agreement with other authors, we found that this implies a diverse diagnostic focus for the detection of these alterations, as well as a potential lack of experience in the diagnosis and classification of the oral mucosa lesions in IBD.^2^ In our results, this factor may significantly contribute to the high detection of oral lesions, considering that the assessments were carried out by oral pathology specialists whose focus was not only to detect those lesions directly associated with IBD or, frankly notorious or symptomatic ones, but all conditions in the oral mucosa.

In addition to the OL classified as specific or non–specific,^2^ other oral conditions such as xerostomia, lichenoid lesions, coated tongue, candidosis, geographic tongue, mucosal abscesses, leukoplakia, erythema, granulomatous cheilitis, bleeding, parodontopathies, together with halitosis, burning sensation, and dysgeusia, have been mentioned as findings in IBD,^2,3,23^ some of which were associated with IBD severity;^24^ nonetheless, most of the published studies of OL in IBD show a moderate to low level of evidence, with considerable disagreement on their classification.^2^ This study reports all mucosal conditions that can be observed in patients with IBD, including those with controlled disease, which can influence morbidity. Nevertheless, notable discrepancies exist in relation to the type and prevalence of oral lesions observed specifically in UC and CD. According to some authors, these are more prevalent in patients with CD.^4,20^ Within our series of patients, we observed a high frequency of oral conditions in both groups, as well as a wide variety of clinical conditions (Table 3), showing important differences in the rates of furred tongue (p = 0.03) and varyx (p = 0.04).

Coated tongue, also known as furred or “furry” tongue, is occasionally misdiagnosed as hairy tongue or pseudomembranous candidosis; and it is popularly believed that a furred tongue in a child is morbid and of clinical significance;^28,29^ however, no relation has been observed between a coated tongue and children’s health status .^28^Nonetheless, alterations in the oral microbiota of a furred tongue have been related to multiple diseases in adults,^29^such as diabetes, obesity, cardiovascular diseases, cancer, and other systemic illnesses. Likewise, tongue–coating microbiota may cause gastritis and digestive system tumors,^30^ fostering the development of chronic diseases. The high frequency of coated tongue observed in our sample confirms its strong association with gastrointestinal tract illnesses.

Our frequency of coated tongue in CD was similar to those in other series (44%);^31^ but greater in our patients with UC (69%) in contrast to other studies (14% and 4.5%).^21,23^The frequency of coated tongue between UC and CD (Table 3) was statistically different even after considering several confounding factors (Table 4), which is a novel finding in IBD. Some authors upon analyzing the presence of coated tongue in individuals with UC did not observe discrepancies compared to their controls,^21,23^ except when contrasting them with severe cases, in which a higher frequency of this oral condition was revealed among individuals with UC receiving pharmacological treatment.^21^ As it is widely known, the combination of multiple risk factors may be involved in the development of tongue–coating microbiota, such as diet, age, immunological status, temperature, humidity, salivary volume and pH, oxygen, and the rate of local mucosal shedding, amongst others. Nonetheless, the mechanism whereby the organism is systemically affected has not been properly elucidated so far, thus warranting broader research into this entity, including a wide spectrum of possible factors in addition to those studied here. These include local and systemic factors,^28^ which may intervene in the development of the entity, and should help explain its potential clinical implications. Hydrogen peroxide rinse is the most effective method for reducing the bacterial count of coated tongue.^32^

Varicosities in IBD are intraoral conditions rarely mentioned in the medical literature. An oral varix (or varicosity) is a common type of acquired vascular malformation, and is regarded as a physiological process of negligible clinical significance, except for those located at sites that are prone to trauma, with the potential for bleeding, or when they are cosmetically objectionable.^33,34^ The prevalence of varicosities ranges from 16.2% to 80%, and an increase in their frequency is observed with increased age, from 10% among young people to 72% in patients older than 70 years.^33^

Varicosities are described in association with several medical diseases, such as venous insufficiency (in relation to lower limb varyx), portal hypertension, chronic vitamin C deficiency,^35^ phlebectasias of jejunum and scrotum,^34^superior vena cava syndrome, chronic hepatitis C, and cirrhosis.^36^In this series, the highest frequency of varicosities in patients with CD in comparison to those with UC was a new finding. This mathematical association disappeared after adjusting the groups for age in the multivariate analysis (Table 4), confirming its previously observed relationship with this variable in other populations.^33,36^This oral condition should be analyzed in future projects to confirm whether this novel finding is related to IBD.

In the medical literature, there is a plethora of descriptions of oral ulcers in IBD, along with a multiplicity of potential pathophysiological mechanisms and known symptoms. This study found a low percentage of ulcerated lesions in the oral mucosa. The most frequent ulcers were of the aphthous type or caused by trauma, which is in agreement with other reports,^11^with reported frequencies ranging from 0.7% to 66%.^23,27^ In fact, aphthous stomatitis can precede IBD diagnosis in 21% of the cases, and it is considered one of the main extraintestinal manifestations of IBD.^5^However, various names have been assigned to a variety of similar ulcers in IBD, e.g., shallow round ulcerations,^4^ recurrent ‘aphthous type’ ulceration,^10^ aphthous–like ulcers,^11^ and aphthoid ulceration.^21^ Another example is pyostomatitis vegetans, whose original description was clinically defined as the development of multiple tiny vegetations with purulent content on an erythematous base.^37,38^These are similar to the milliar abscesses observed in the “pyodermatite végétante” of Hallpeau^39^ in synchrony with gastrointestinal symptoms.^38^This condition, however, has lost its clinical “vegetans” essence, now being referred to as mere ulcers of the oral mucosa with an unspecified histological nature.^40^ As a consequence, we have a plurality of entities described in IBD probably referring to the same condition, or which might correspond to other type of oral conditions, thus potentially generating diagnostic errors and difficulties in their study and teaching.

Of note, most oral ulcers in patients with IBD appear to arise from friction on a generally atrophic mucosa in the presence of xerostomia, chemical burns (e.g. topical substances for oral hygiene or irritating foods in the daily diet) or inflammation caused by candidosis, to mention just a few causes. The diagnosis of traumatic ulcers in such cases may not be easily established either clinically or histologically by an untrained clinical eye. Atrophy tends to be the sine qua non in cases of oral ulcers in IBD, consistent with a high frequency of atrophic glossitis, xerostomia, and candidosis (40%, 42%, and 70%, respectively), among other common predisposing factors for the development of ulcers or traumatic lesions, as in this study. However, these associations have been scarcely analyzed in epidemiological studies concerning the etiology and prevalence of OL in IBD.

Microscopically, traumatic ulcers correspond mainly to a mixed inflammatory process of neutrophilic predominance compatible with that observed in recurrent aphthous stomatitis,^41^ ulceration not otherwise specified in HIV,^42^ those related to nutritional diseases, and Behçet disease,^43^ and even compatible with the recent descriptions referred to as pyostomatitis vegetans,^44^ to mention a few. This is why the histological diagnosis of an oral ulcer in IBD based exclusively on a nonspecific mixed inflammatory response could be wrong. Nonetheless, the clinical and serological characteristics of ulcers in IBD differ widely among patients; therefore, in these cases, oral examination by experts using a multidisciplinary approach is warranted, as suggested,^21,27^ highlighting the importance of evaluating the patient via a careful clinical history, examination, and laboratory and cabinet tests. This profound diagnostic exercise aims to rule out the diverse etiological possibilities, thereby providing the patient with high–quality care.^27^

In our sample, the high rates of oral mucosa atrophy in IBD may be largely attributed to a frequent decrease in serum micronutrients such as folate, vitamin B_12_, 25–OH–vitamin D, and ferritin, which is commonly seen in this type of patients.^45^ In our findings, a higher frequency of recurrent aphthous ulcers was observed in patients with CD, coinciding with lower hemoglobin levels compared to UC patients (Table 2). We could also observe (Table 2) that many patients had fat and muscle mass depletion (as indicated by a low triceps skinfold thickness and arm muscle area, respectively). In line with these findings, the presence of oral ulcers in IBD patients increases as the disease worsens,^21^perhaps in relation to the low serum iron and folate concentrations particularly found in CD patients;^46^ thus, we recommend additional surveillance of these nutrients and their supplementation in case of deficiency. Immunological mechanisms may also have an influence on the etiology of mouth ulcers in IBD.^19^ Nonetheless, it is important to consider that the development of oral mucosal lesions in IBD patients involves not only nutritional deficiencies, but other variables such as infections, drug side effects, and other inflammatory conditions as well.

Notably, recent reviews of the literature have shown that nutritional deficiencies are related to the development of lesions in the oral mucosa, such as atrophic glossitis, xerostomia, or burning mouth, in different clinical scenarios; however, this has been scarcely studied in IBD. ^10,25,26^ Our work stands out for its exceptional approach regarding the combined evaluation of the nutritional and serological status associated with each of the multiple oral findings in this subset of IBD patients, and taking into account that these patients commonly have nutritional deficiencies. In our findings, although no statistical differences were observed between the presence of various OLs and serological values of the studied nutrients, lower levels were observed for most nutrients (Table 5). Likewise, we underscore that, in our findings, the presence of mucosal pallor was a common oral finding (Table 3) and it was closely associated with nutrition, but it has rarely been mentioned in the IBD literature.

In this study, the frequency of melanosis, an entity rarely reported in IBD, was prevalent in our mestizo population sample. To our knowledge, the observed association with the use of antiseptic mouthwashes has not been previously mentioned,^47^ but this information raises several questions, including whether patients with IBD and mestizo phototypes are more susceptible to developing melanosis from the use of mouth rinses or whether there are other causes of melanosis concurrently with the use of mouth rinses. Such questions need to be investigated more profoundly in future research and analyze variables such as the reasons of use of oral rinses, the type of rinses, frequency and time of use, additional comorbidities, use of drugs, etc.

Finally, to interpret our results, some limitations should be considered, such as the reduced number of patients and the lack of a control group (without IBD). While a statistical analysis was performed on all included variables, it is difficult to determine the effects of certain factors on the development of OL, such as the administration of IBD treatment, among other factors. Further research including other variables that may contribute to the development of OL, such as comorbidities in IBD, serum levels of several micronutrients, parafunctional habits, amount of saliva production, etc, is warranted.

In brief, in this study, we established and analyzed the frequency of OL observed in patients with IBD, including the multivariate analysis of nutritional status, certain serological parameters, and local and clinical variables related to IBD. This approach can broaden the knowledge of physicians regarding OLs and promote their prompt detection and management.

## Conclusions

Every participant in this study presented one or more OLs. Type of IBD, older age, sex, low levels of hemoglobin, use of mouthwashes, smoking, and candidosis were predictive factors of OL in these patients; therefore, it is important to become acquainted with OL in IBD because some may exacerbate the underlying condition or interfere with patient quality of life or with the treatment. Numerous clinical descriptions on oral lesions in IBD have been published; thus, it is imperative that future studies consider more rigorous diagnostic criteria with the aim of deepening our knowledge of these conditions.

## Data Availability

The contents underlying the research text are contained in the manuscript.

## References

[1] Ozer M, Bengi G, Colak R, Cengiz O, Akpinar H (2020). Prevalence of irritable bowel syndrome-like symptoms using Rome IV criteria in patients with inactive inflammatory bowel disease and relation with quality of life. Medicine (Baltimore).

[2] Lauritano D, Boccalari E, Di Stasio D, Della Vella F, Carinci F, Lucchese A (2019). Prevalence of oral lesions and correlation with intestinal symptoms of inflammatory bowel disease: a systematic review. Diagnostics (Basel).

[3] Lourenço SV, Hussein TP, Bologna SB, Sipahi AM, Nico MM (2010). Oral manifestations of inflammatory bowel disease: a review based on the observation of six cases. J Eur Acad Dermatol Venereol.

[4] Ribaldone DG, Brigo S, Mangia M, Saracco GM, Astegiano M, Pellicano R (2020). Oral manifestations of inflammatory bowel disease and the role of non-invasive surrogate markers of disease activity. Medicines (Basel).

[5] Jose FA, Garnett EA, Vittinghoff E, Ferry GD, Winter HS, Baldassano RN (2009). Development of extraintestinal manifestations in pediatric patients with inflammatory bowel disease. Inflamm Bowel Dis.

[6] Mowat C, Cole A, Windsor A, Ahmad T, Arnott I, Driscoll R (2011). Guidelines for the management of inflammatory bowel disease in adults. Gut.

[7] Trost LB, McDonnell JK (2005). Important cutaneous manifestations of inflammatory bowel disease. Postgrad Med J.

[8] Mariela Torres I, Paz K, Salazar FG Tamaño de una muestra para una investigación de mercado.

[9] Botella-Rocamora P, Alacreu-García M, Martínez-Beneito MA Inferencia estadística (intervalos de confianza y p-valor). Comparación de dos poblaciones (test t de comparación de medias, comparación de dos proporciones, comparación de dos varianzas).

[10] Asquith P, Thompson RA, Cooke WT (1975). Oral manifestations of Crohn's disease. Gut.

[11] Zervou F, Gikas A, Merikas E, Peros G, Sklavaina M, Loukopoulos J (2004). Oral lesions in patients with inflammatory bowel disease. Ann Gastroenterol.

[12] Kramer IR, Pindborg JJ, Bezroukov V, Infirri JS (1980). Guide to epidemiology and diagnosis of oral mucosal diseases and conditions. Community Dent Oral Epidemiol.

[13] Ristevska I, Armata RS, D'Ambrosio C, Furtado M, Anand L, Katzman MA (2015). Xerostomia: understanding the diagnosis and the treatment of dry mouth. J Fam Med Dis Prev.

[14] Callen JP, Jorizzo JL, Bolognia JL, Piette WW, Zone JJ (2009). Dermatological signs of internal disease.

[15] Truelove SC, Witts LJ (1955). Cortisone in ulcerative colitis; final report on a therapeutic trial. BMJ.

[16] Keyashian K, Dehghan M, Sceats L, Kin C, Limketkai BN, Park KT (2019). Comparative incidence of inflammatory bowel disease in different age groups in the United States. Inflamm Bowel Dis.

[17] Hong SW, Park J, Yoon H, Yang HR, Shin CM, Park YS (2021). Comparison of loss of response between anti-tumor necrosis factor alone and combined use with immunomodulators in patients with inflammatory bowel disease. Korean J Intern Med (Korean Assoc Intern Med).

[18] Jess T, Loftus EV, Harmsen WS, Zinsmeister AR, Tremaine WJ, Melton LJ (2006). Survival and cause specific mortality in patients with inflammatory bowel disease: a long term outcome study in Olmsted County, Minnesota, 1940-2004. Gut.

[19] Lankarani KB, Sivandzadeh GR, Hassanpour S (2013). Oral manifestation in inflammatory bowel disease: a review. World J Gastroenterol.

[20] Klichowska-Palonka M, Komsta A, Pac-Kozuchowska E (2021). The condition of the oral cavity at the time of diagnosis of inflammatory bowel disease in pediatric patients. Sci Rep.

[21] Elahi M, Telkabadi M, Samadi V, Vakili H (2012). Association of oral manifestations with ulcerative colitis. Gastroenterol Hepatol Bed Bench.

[22] Harty S, Fleming P, Rowland M, Crushell E, McDermott M, Drumm B (2005). A prospective study of the oral manifestations of Crohn's disease. Clin Gastroenterol Hepatol.

[23] Kumar KM, Nachiammai N, Madhushankari GS (2018). Association of oral manifestations in ulcerative colitis: a pilot study. J Oral Maxillofac Pathol.

[24] Tabarsi NT, Mortazavi N, Norouzi A, Besharat S, Behnampour N, Asgari N (2024). Association of oral manifestations with severity of the disease in ulcerative colitis patients. BMC Gastroenterol.

[25] Pecci-Lloret MP, Ramirez-Santisteban E, Hergueta-Castillo A, Guerrero-Gironés J, Oñate-Sánchez RE (2023). Oral manifestations of Crohn's disease: a systematic review. J Clin Med.

[26] Li C, Wu Y, Xie Y, Zhang Y, Jiang S, Wang J (2022). Oral manifestations serve as potential signs of ulcerative colitis: a review. Front Immunol.

[27] Sbeit W, Kadah A, Mahamid M, Karayanni H, Mari A, Tali S (2020). Oral manifestations of inflammatory bowel disease: the neglected piece of the puzzle. Eur J Gastroenterol Hepatol.

[28] Gans B (1954). The fallacy of the furred tongue. BMJ.

[29] Sun S, Wei H, Zhu R, Pang B, Jia S, Liu G (2018). Biology of the tongue coating and its value in disease diagnosis. Complement Med Res.

[30] Cui J, Cui H, Yang M, Du S, Li J, Li Y (2019). Tongue coating microbiome as a potential biomarker for gastritis including precancerous cascade. Protein Cell.

[31] Katz J, Shenkman A, Stavropoulos F, Melzer E (2003). Oral signs and symptoms in relation to disease activity and site of involvement in patients with inflammatory bowel disease. Oral Dis.

[32] Soutome S, Otsuru M, Hayashida S, Naruse T, Morishita K, Kurihara K (2022). Efficacy of 3% hydrogen peroxide solution in cleaning tongue coating before and after surgery: a randomized phase II study. BMC Oral Health.

[33] Lazos JP, Piemonte ED, Panico RL (2015). Oral varix: a review. Gerodontology.

[34] Rappaport I, Shiffman MA (1964). The significance of oral angiomas. Oral Surg Oral Med Oral Pathol.

[35] Eddy TP, Taylor GF (1977). Sublingual varicosities and vitamin C in elderly vegetarians. Age Ageing.

[36] Duarte NT, Godoy OA, Tenório JR, Andrade NS, Franco JB, Pérez-Sayáns M (2020). Prevalence of sublingual varices in patients with cirrhosis and the correlation with nitrogen compounds. Oral Surg Oral Med Oral Pathol Oral Radiol.

[37] Wallhauser HJ (1929). Dermatitis vegetans: report of 2 cases of the Hallopeau type. Arch Derm Syphilol.

[38] Goldsmith WN (1941). A case of pyodermite végétante (Hallopeau). Br J Dermatol.

[39] Hallopeau H (1898). Pyodermite végétante", ihre Beziehungen zur Dermatitis herpetiformis und dem Pemphigus vegetans. Arch Dermatol Res.

[40] Petruzzi M, Della Vella F (2021). Pyostomatitis vegetans. N Engl J Med.

[41] Gasmi Benahmed A, Noor S, Menzel A, Gasmi A (2021). Oral aphthous: pathophysiology, clinical aspects and medical treatment. Arch Razi Inst.

[42] Prabhu RV, Prabhu V, Chatra L, Shenai P (2013). Oral manifestations of HIV. J Trop Dis.

[43] Lehner T (1969). Pathology of recurrent oral ulceration and oral ulceration in Behcet's syndrome: light, electron and fluorescence microscopy. J Pathol.

[44] Hegarty AM, Barrett AW, Scully C (2004). Pyostomatitis vegetans. Clin Exp Dermatol.

[45] Park YE, Park SJ, Park JJ, Cheon JH, Kim T, Kim WH (2021). Incidence and risk factors of micronutrient deficiency in patients with IBD and intestinal Behçet's disease: folate, vitamin B12, 25-OH-vitamin D, and ferritin. BMC Gastroenterol.

[46] Dawson AM (1972). Nutritional disturbances in Crohn's disease. Br J Surg.

[47] Sreeja C, Ramakrishnan K, Vijayalakshmi D, Devi M, Aesha I, Vijayabanu B (2015). Oral pigmentation: a review. J Pharm Bioallied Sci.

